# Supplementary material for: Exploring the role of temperature and other environmental factors in West Nile virus incidence and prediction in California counties from 2017–2022 using a zero-inflated model
Source: PLoS Negl Trop Dis. 2024 Jun 24;18(6):e0012051. doi: 10.1371/journal.pntd.0012051 (PMC11226093; doi:10.1371/journal.pntd.0012051)
Supplement: Attachment — Submitted filename: Revision_Letter.docx [file pntd.0012051.s001.docx]

Noah Parker

[noahtparker3@berkeley.edu](mailto:noahtparker3@berkeley.edu)

June 1, 2024

To The Editorial Staff at PLOS Neglected Tropical Diseases,

Thank you for taking the time and effort to review my manuscript. I appreciate the comments left by the peer reviewers and have revised my work where applicable. Below is a list of my responses to the peer review comments and whether I revised my manuscript to account for them.

- Reviewer #1 expressed a lack of clarity in Table 1. After further consideration, I have decided to remove the table from the paper completely. We believe that Figure 1 already shows the important parts of Table 1: cases and temperature across time. It doesn’t appear that Table 1 adds much to the paper and may further confuse readers. If the editorial staff believes that Table 1 is beneficial to the understanding of the paper, I can add it as supplemental material, after revising it to add more clarity.
- Reviewer #2 asked for more common English explanations of the two types of models. In the introduction section, we added a quick explanation of the reasons for using a zero-inflated model generally. In the methods section, a longer, non-statistical explanation was added for both the zero-inflated and GEE models. Finally, to make it more clear that a goal of this paper was to compare the zero-inflated model against the GEE model, we added a short hypothesis about comparing the two (in the introduction section) and added “using a zero-inflated model” to the title.
- Reviewer #2 expressed concerns about the time period restrictions. The original manuscript stated that the six year period “reflected a tradeoff between having a large number of observations while maintaining a sufficiently contained time range where transmission and meteorological conditions expressed similar characteristics.” To further clarify this point, we added information about the trends of statewide average monthly temperatures over the last 15 years. The data source used for this was added to the reference list at #11. We also ran a sensitivity analysis by using different sets of years in our measure of association test — one dataset having all years except 2017, another having all years except 2019, etc. All four of the adjusted datasets, including the one with only four years of data, estimated an IRR in the zero-inflated model within 2% of the IRR using the six year dataset, leading us to believe that the time period didn’t have a significant effect on our results. These revisions were added to the Methods I. Data section.
- Reviewer #2 asked if we had considered other temperature increases. We appreciated this idea and incorporated into our new manuscript. We created a new figure, now Fig 3, to highlight the results of this analysis. A quick description is provided above the figure in the results section, and the standardization methods were updated to include the other increases in temperature.
- Reviewer #2 posed if we had considered humidity in our analysis. While we had considered humidity, we chose not to include it in our paper for two reasons. First, humidity data was not available from the NOAA stations from where we received temperature and precipitation data. Second, and more importantly, other papers in the literature that conducted similar analyses with temperature also did not use humidity data. We therefore believe this omission is warranted.
- Reviewer #2 expressed some concerns over non-scientific language. We went back through and corrected language throughout — both the places where the reviewer expressed concern and other places where we thought our language could be more precise and scientific. The changes are highlighted throughout the tracked changes document.

Thank you again for your consideration. Please let me know if you have further questions or concerns.

Sincerely,

Noah Parker
